# Depression Disparities in Older Korean and Chinese Immigrants in the United States

**DOI:** 10.1093/geroni/igaf122.3417

**Published:** 2025-12-31

**Authors:** Ruimin Yang, Jeongone Seo, Shirley Qiu, Gracie Shao

**Affiliations:** Columbia University, Teachers College, New York, New York, United States; Sungkyukwan University, Seoul, Republic of Korea; University of California, Santa Barbara, Santa Barbara, California, United States; The University of British Columbia, Vancouver, British Columbia, Canada

## Abstract

This meta-analysis compares depression levels among Chinese and Korean immigrants aged 60 and older in the United States. Studies published between 2010 and 2024 were synthesized using standardized mean differences (SMDs) from a random-effects model. Korean immigrants reported higher depressive symptoms than Chinese immigrants when assessed with the Patient Health Questionnaire (PHQ) (SMD = 0.0778, SE = 0.0193, p < .0001, 95% CI [0.0409, 0.1147]), but no significant difference emerged with the Geriatric Depression Scale (GDS) (SMD = -0.0060, SE = 0.0625, p = 0.9233, 95% CI [-0.1284, 0.1164]). Within-group analyses indicated that GDS produced higher depression scores than PHQ for both groups. Among Chinese immigrants, PHQ scores were significantly lower than GDS scores (SMD = -0.2430, SE = 0.0411, p < .0001, 95% CI [-0.3235, -0.1625]), suggesting PHQ may underestimate depression. Likewise, Korean immigrants recorded lower PHQ scores than GDS scores (SMD = -0.1601, SE = 0.0569, p = 0.0049, 95% CI [-0.2715, -0.0486]). Korean immigrants’ CES-D scores were also lower than GDS scores (SMD = -0.0998, SE = 0.0417, p = 0.0167, 95% CI [-0.1816, -0.0181]). Potential contributors included acculturation, social support, socioeconomic status, language barriers, healthcare availability, and intergenerational dynamics. These findings underscore the importance of culturally tailored screening and interventions. Research should address measurement disparities, investigate longitudinal patterns, and develop targeted strategies to reduce depression among older Asian immigrants. This could inform policies and optimize mental health outcomes for this vulnerable population. Clinicians and policymakers should prioritize culturally responsive mental health screening, interventions, and policy reforms.

